# Myopericarditis in a Young Adult Secondary to COVID-19 Vaccination

**DOI:** 10.14797/mdcvj.847

**Published:** 2021-09-22

**Authors:** Corbin G. Walters, Dev D. Jaiswal, Tom X. Hu, Steve S. Kim

**Affiliations:** 1 Oklahoma State University Center for Health Sciences, Tulsa, Oklahoma, US; 2 Oklahoma State University Medical Center, Tulsa, Oklahoma, US

**Keywords:** COVID-19, SARS-CoV-2, vaccination, echocardiography, myocarditis, pericarditis

## Abstract

We present the case of a healthy 29-year-old male with no significant medical history who presented with electrocardiogram findings consistent with pericarditis and elevated troponin levels, commonly seen in myocarditis, after receiving his second Pfizer-BioNTec vaccination for SARS-CoV-2 (severe acute respiratory syndrome coronavirus 2). The patient had significant clinical improvement shortly after receiving aspirin and colchicine and was discharged home with these medications. His laboratory findings returned to baseline less than 2 weeks after his illness. While this case highlights the importance of diagnosis, intervention selection, and treatment of myopericarditis amid ongoing global vaccination campaigns, it should be emphasized that the benefits of vaccination considerably outweigh the risks.

## Background

Vaccination for SARS-CoV-2 (severe acute respiratory syndrome coronavirus 2) has given much of the world a welcome respite after the disease caused more than 3.5 million deaths worldwide over the last 18 months.^1^ Mass vaccination campaigns across the globe have resulted in nearly 3 billion doses given by June 2021, which has dramatically decreased the number of cases and deaths. As more individuals receive vaccines, increased reports of adverse events secondary to vaccination may appear. A growing concern among the public is the development of myopericarditis, an exceedingly rare event reported primarily in adolescent males receiving vaccines that use mRNA technology. This finding has spurred the US Food and Drug Administration (FDA) to add a warning for myocarditis to mRNA vaccines. However, little is known about the development of myopericarditis secondary to vaccination in adults.

Pericarditis is the inflammation of the pericardium, commonly caused by viral and other infectious sources. It is responsible for approximately 0.2% of all hospitalizations and 5% of patients admitted from the emergency department (ED) for chest pain in the absence of ischemia.^2,3^ SARS-CoV-2 is a rapidly spreading and aggressive respiratory virus responsible for multisystem inflammatory dysfunction, including respiratory failure, ischemic stroke, pulmonary embolism, myocardial infarction, and other complications, many of which are fatal.^4^ Myocardial injury, such as myocarditis and pericarditis, is a rare presentation of SARS-CoV-2.^5^ However, myocardial injury secondary to SARS-CoV-2 vaccination in the absence of SARS-CoV-2 infection has recently become an area of concern among clinicians and the public. More than 171 million individuals in the United States have received at least one vaccination for SARS-CoV-2, with more than 96% of these vaccinations developed by Pfizer-BioNTec and Moderna.^6^ Despite the vaccines’ celebrated efficacy and favorable side effect profile, rare adverse events are being reported and widely circulated. Although case reports are available examining myocardial injury secondary to vaccination, most cases have been seen in young adults and adolescents.^7^ As of April 2021, more than 1,000 cases of myocarditis secondary to vaccination had been reported to the Centers for Disease Control and Prevention (CDC), prompting the FDA to add a warning to the Pfizer and Moderna vaccines notifying recipients of the rare but concerning adverse event.^8^ With this case report, we seek to contribute to the limited available literature for the diagnosis and treatment of myopericarditis after SARS-CoV-2 vaccination in young adults.

## Case Report

A 29-year-old male with no past medical history presented to the emergency department (ED) with chest pain for the past day. The patient initially attributed the pain to gastroesophageal reflux but failed to alleviate his symptoms with over-the-counter medications. Over several hours, the patient reported that the vague chest pain became sharp, substernal, and without radiation, prompting him to go to the ED.

Once there, the patient was hemodynamically stable and afebrile, with a blood pressure of 122/75 mm Hg, heart rate of 61 beats per minute, respiratory rate of 16 breaths per minute, and oxygen saturation of 99% on room air. He reported symptoms of dyspnea, nausea, and substernal chest pain. He stated that he was in good health, required no medications, and recieved his second Pfizer-BioNTec vaccination approximately 4 days before the onset of chest pain. He denied a history of recent illness, fevers, chills, palpitations, diaphoresis, syncope, or a ripping or tearing sensation in his chest. He has no history of thromboembolism, hypercoagulability disorders, recent surgery, nor periods of immobilization. The patient also denied tobacco and illicit drug use or a family history of coronary artery disease. The physical examination was unremarkable, and a cardiac exam revealed a regular rate and rhythm without murmurs, clicks, or rubs. A pulmonary exam revealed equal breath sounds auscultated bilaterally.

**Figure 1:**
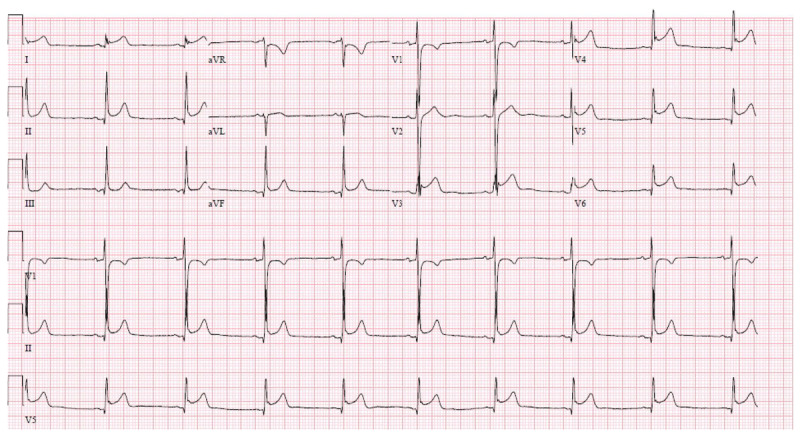
Initial electrocardiogram upon arrival.

A chest x-ray showed no acute processes and an unremarkable mediastinum. Laboratory results from the comprehensive metabolic panel and complete blood count with differential were within normal limits. Serum creatinine kinase was 386 U/L (normal = 39–308 U/L) and troponin 5.97 ng/mL (normal ≤ 0.04 ng/mL). Twelve-lead electrocardiogram (ECG) reported sinus bradycardia with a rate of 56 beats per minute, normal axis of deviation, PR interval of 128 ms (normal = 120–200 ms), QRS complex duration of 98 ms (normal = 80–100 ms), QTC interval of 370 ms (normal ≤ 440 ms), and diffuse ST segment elevation throughout (***Figure 1***). The findings of the ECG suggested pericarditis. Given the patient’s elevated cardiac markers and abnormal ECG, the cardiology team was consulted and the patient was admitted to the general medical floor.

**Figure 2:**
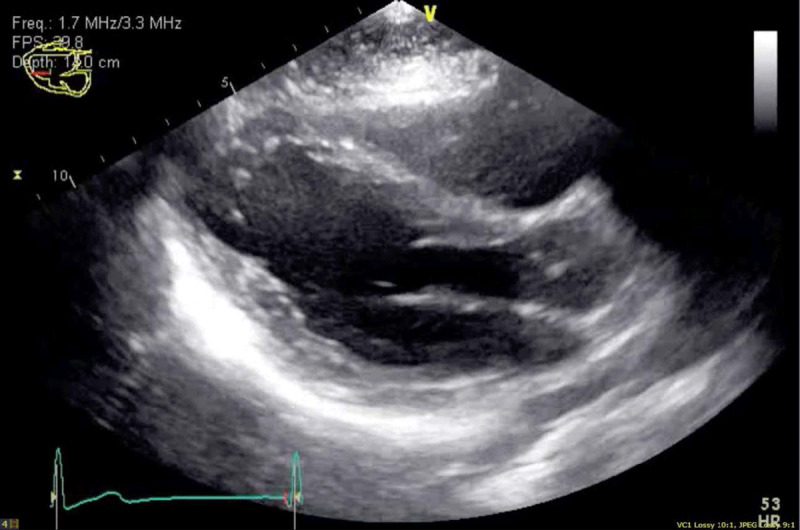
Transthoracic echocardiogram with a parasternal long-axis view that demonstrates a preserved left ventricular ejection fraction with no pericardial effusion.

**Figure 3:**
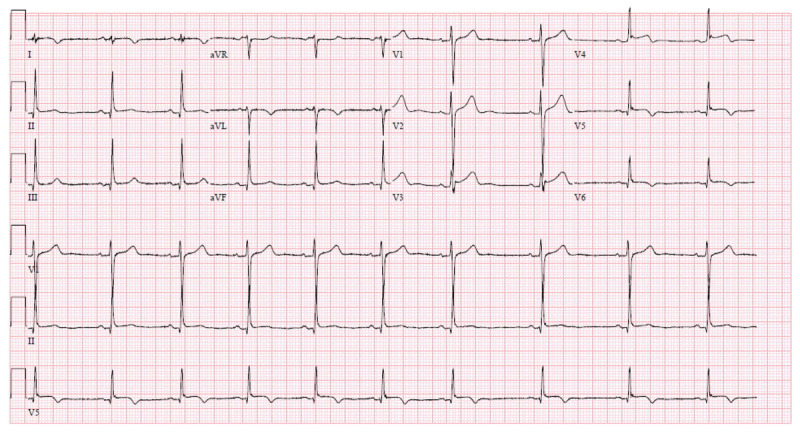
Subsequent electrocardiogram after initiation of treatment.

Myopericarditis was strongly suggested based on patient presentation, findings of the ECG, and elevated troponin. Other potential diagnoses in the differential included myocarditis, cardiac injury, and ST-elevated myocardial infarction. The patient underwent transthoracic echocardiography (TTE), which revealed no wall motion abnormalities, normal left ventricular diastolic function, and an estimated ejection fraction of 60% (***Figure 2***, ***Videos 1^2^,3***). No effusion was noted. The patient’s troponin peaked at 13.47 ng/mL. Subsequent ECGs revealed improvement in diffuse ST segment elevation (***Figure 3***).

**Video 1:** Transthoracic echocardiogram showing a parasternal long-axis view. *https://youtu.be/_UGS9V5PvoE*

**Video 2:** Transthoracic echocardiogram showing an apical four-chamber view. *https://youtu.be/K1s9sI8ckvQ*

**Video 3:** Transthoracic echocardiogram showing an apical two-chamber view. *https://youtu.be/C5lkhG20SoY*

Given the patient’s history of recent vaccination with supporting laboratory and ECG findings, myopericarditis secondary to SARS-CoV-2 vaccination was suspected. However, a direct causal relationship between vaccination and development of myopericarditis cannot be inferred based on this case presentation. Rather, this clinical diagnosis was made with suspicion of myopericarditis given circulating case reports and the absence of other aggravating factors. The patient received 162 mg of aspirin in the emergency department. He was started on 325 mg of aspirin three times daily and 0.6 mg colchicine twice a day for 1 day, with 0.6 mg once a day afterward because he weighed less than 70 kilograms. The patient reported immediate improvement in his symptoms.

The patient was discharged in stable condition 1 day after his admission. He was instructed to take 325 mg aspirin 3 times a day for 3 months and 0.6 mg colchicine once per day for 3 months. The patient was seen by his primary care physician approximately 12 days after discharge and reported complete resolution of his symptoms. Laboratory results performed at the appointment revealed a return to baseline for all markers, including serum creatine kinase and troponin. The patient was seen in the cardiovascular medicine clinic approximately 70 days after discharge and reported complete resolution of his symptoms.

## Discussion

Myopericarditis is an exceedingly rare yet not unprecedented adverse event secondary to vaccination. Similar findings have been reported for individuals vaccinated for smallpox, measles, varicella, polio, and other diseases.^9,10^ However, the use of novel mRNA vaccine technology and the addition of a warning for heart inflammation has become a source of public misinformation and subsequent vaccine hesitancy despite the overwhelming success of these vaccines in preventing hospitalization and death from SARS-CoV-2 infection.

A report by the CDC Advisory Committee for Immunizations found 1,226 cases of myopericarditis between December 29, 2020, and June 11, 2021, occurring most often in young men (median age of 26 years), typically 3 days after receiving their second vaccination.^11^ Although the mechanism of injury is unknown, it is believed to result from an autoimmune reaction,^12^ which suggests a potentially indirect cardiac injury by the immune system in response to vaccination rather than direct injury from the vaccine components. Future research is warranted to investigate the underlying pathophysiology responsible for vaccine-induced myopericarditis and the predilection to affect males after the second dose. Individuals with vaccine-induced myopericarditis commonly report acute onset of chest pain, decreased exercise tolerance, and dyspnea, and symptoms resolve completely once nonsteroidal anti-inflammatory drugs and supportive treatment are administered.^11^ Along with exercise restriction, these treatment considerations are evidence-driven treatments for myopericarditis,^3^ suggesting that this approach can be similarly applied to individuals with myopericarditis caused by the SARS-CoV-2 vaccine.

## Conclusion

The case of myopericarditis in a young adult male with no previous medical history began shortly after receiving his second vaccination for SARS-CoV-2 infection. This rare but debilitating disease is a cause of concern for the public because mass vaccination campaigns for SARS-CoV-2 are ongoing throughout much of the world, resulting in increased reports of vaccine-attributed adverse events. Therefore, educating clinicians on the identification, presentation, and treatment of myopericarditis and supporting future research efforts are of the utmost importance. However, it should be emphasized that despite these rare reports of myopericarditis, the benefits of vaccination considerably outweigh the risks.
